# Sodium-Glucose Cotransporter-2 Inhibitors for Hyperglycemia in Phosphoinositide 3-kinase Pathway Inhibition

**DOI:** 10.21203/rs.3.rs-2655905/v1

**Published:** 2023-03-21

**Authors:** Michael A Weintraub, Dazhi Liu, Raymond DeMatteo, Marcus DaSilva Goncalves, James Flory

**Affiliations:** NYU Langone Medical Center: NYU Langone Health; Memorial Sloan Kettering Cancer Center; Memorial Sloan Kettering Cancer Center; Weill Cornell Medical College: Weill Cornell Medicine; Memorial Sloan Kettering Cancer Center

**Keywords:** alpelisib, hyperglycemia, diabetic ketoacidosis, PIK3CA, metastatic breast cancer, adverse effects

## Abstract

**Purpose:**

Phosphoinositide 3-kinase (PI3K) inhibition is used for the treatment of certain cancers, but can cause profound hyperglycemia and insulin resistance, for which sodium-glucose cotransporter-2 (SGLT2) inhibitors have been proposed as a preferred therapy. The objective of this research is to assess the effectiveness and safety of SGLT2 inhibitors for hyperglycemia in PI3K inhibition.

**Methods:**

We conducted a single-center retrospective review of adults initiating the PI3k inhibitor alpelisib. Exposure to different antidiabetic drugs and adverse events including diabetic ketoacidosis (DKA) were assessed through chart review. Plasma and point-of-care blood glucoses were extracted from the electronic medical record. Change in serum glucose and the rate of DKA on SGLT2 inhibitor versus other antidiabetic drugs were examined as co-primary outcomes.

**Results:**

We identified 103 patients meeting eligibility criteria with median follow-up of 85 days after starting alpelisib. When SGLT2 inhibitors were used to treat hyperglycemia, they were associated with a decrease in mean random glucose by −54 mg/dL (95% CI −99 to −8) in adjusted linear modeling. Five cases of DKA were identified, two occurring in patients on alpelisib plus SGLT2 inhibitor. Estimated incidence of DKA was: alpelisib plus SGLT2 inhibitor, 24 DKA cases per 100 patient-years (95% CI 6, 80); alpelisib with non-SGLT2 inhibitor antidiabetic drugs, 7 (95% CI 0.1, 34); alpelisib only, 4 (95% CI 0.1, 21).

**Conclusions:**

SGLT2 inhibitors are effective treatments for hyperglycemia in the setting of PI3K inhibition, but given possible adverse events, SGLT2 inhibitors should be used with caution.

## Introduction

The phosphoinositide 3-kinase (PI3K) pathway is critically important for tumor progression–promoting cell growth, motility, survival, and metabolism. In breast cancer, the PI3K pathway can be hyperactivated by somatic mutations in *PIK3CA*, which encodes the catalytic subunit of PI3K (P110α). These mutations are found in 40% of breast tumors, and associate with resistance to conventional anticancer agents [1]. Tumor PI3K signaling can be therapeutically targeted by small molecules that specifically bind and inhibit P110α-like taselisib, serabelisib, inavolisib, and alpelisib. Alpelisib was shown to improve clinical outcomes in postmenopausal women and men with hormone receptor-positive, HER2-negative, *PIK3CA*-mutant advanced breast cancer in the SOLAR-1 trial (Study Assessing the Efficacy and Safety of Alpelisib Plus Fulvestrant in Men and Postmenopausal Women With Advanced Breast Cancer Which Progressed on or After Aromatase Inhibitor Treatment [NCT02437318])[2].

In addition to blocking the action of mutant PI3K in the tumor, PI3K inhibitors systemically inhibit PI3K in host tissues, which impairs the intracellular action of insulin and induces insulin resistance. PI3K inhibitor driven insulin resistance causes hyperglycemia, which can be severe [3, 4]. Of 284 patients who received alpelisib in SOLAR-1, hyperglycemia occurred in 63.7% of patients, with fasting plasma glucose levels exceeding 250 mg/dL in 36.6%[2]. Severe hyperglycemia caused by alpelisib has also led to diabetic ketoacidosis (DKA) in some case reports [5–7]. These on-target adverse hyperglycemic events were reported in other trials of P110α inhibitors, as well as inhibitors of AKT, another targetable kinase in the PI3K signaling cascade [8].

Hyperglycemia related to systemic inhibition of PI3K and AKT can result in emergency room visits, inpatient admissions, treatment interruptions, dose reductions, and treatment discontinuation. In a retrospective study of 251 cancer patients new to PI3Kα inhibitor use, we found hyperglycemia caused dose interruption in 13%, dose reduction in 11%, and hospitalization in 3% of patients [9]. Of note, this population largely consisted of clinical trial participants for whom baseline type 1 or 2 diabetes was an exclusion criterion. In ‘real world’ settings, where patients with diabetes are not excluded, hyperglycemia may be a major challenge to using PI3K or AKT inhibitors. Approaches to hyperglycemia management in patients who receive alpelisib and other PI3K/AKT inhibitors are not clearly established. Blocking the PI3K/AKT pathway can induce a highly insulin resistant phenotype in which some antidiabetic drug classes, particularly insulin and insulin secretagogues, might be ineffective. In SOLAR-1, the study protocol was amended about half-way through enrollment to encourage the use of metformin as a first-line anti-hyperglycemic agent which resulted in its use in 87.1% of patients with hyperglycemia [10]. However, the effectiveness of metformin in the setting of PI3K inhibitor use has still not been systematically studied and the effects of other antidiabetic drug classes in this setting are even less well-understood. Choice of an antidiabetic agent in the setting of PI3K/AKT inhibitor use may have implications beyond the quality of glycemic control. For example, high levels of endogenous or exogenous insulin might overcome the therapeutic blockade of PI3K and limit the anti-cancer efficacy, as has been shown in animal models [3].

Metformin is recommended as a first-line agent for the treatment of alpelisib-induced hyperglycemia, even though its effectiveness in the setting of PI3K blockade is not established [10]. Of alternative antidiabetic drug classes, sodium-glucose cotransporter-2 (SGLT2) inhibitors have received special attention. In animal models with cancer exposed to PI3K inhibitors, SGLT2 inhibitors reduced glucose and insulin levels more than metformin and intriguingly were also associated with reduced cancer progression [11–13]. In humans, several clinical case reports describe impressive glycemic responses to SGLT2 inhibitors in patients on alpelisib [3, 14, 15]. In our prior exploratory analysis we found SGLT2 inhibitor use was associated with larger glucose reductions than other classes of antidiabetic drugs (e.g., metformin) [9]. However, SGLT2 inhibitors are not without risk. We and others have identified cases of DKA in patients receiving alpelisib and SGLT2 inhibitors [5, 9, 16]. SGLT2 inhibitor therapy increases the risk for euglycemic DKA, which could be exacerbated by alpelisib-induced insulin resistance. Therefore, we conducted this follow-up retrospective cohort study with two goals: to test the hypothesis that SGLT2 inhibitors reduce glucose more effectively than other classes of antidiabetic drugs in patients on alpelisib, and to describe the rate of DKA with and without SGLT2 inhibitor use in alpelisib-treated patients.

## Research Design And Methods

This is a single-center retrospective review of patients aged > 18 years initiating alpelisib treatment at a tertiary cancer center, Memorial Sloan Kettering (MSK). The research protocol was approved by MSK’s Institutional Review Board. Patients were potentially eligible if they initiated alpelisib on any date prior to May 26, 2022. To ensure that findings of the current work were not duplicative of a previous publication, patients were excluded if any of their data were included in that publication [9]. No exclusion criteria by cancer type, or stage were imposed, although it was expected that nearly all patients received alpelisib according to its clinical indication for metastatic breast cancer in patients with activating PI3K mutations.

Alpelisib users were identified by a database query for electronic prescriptions for alpelisib. Alpelisib use was confirmed by manual chart review by two reviewers (any of MAW, DL, RD, JF). Baseline comorbidities, height, weight, body mass index (BMI, in kg/m^2^), random serum and point-of-care glucoses, serum creatinine, serum albumin, serum bicarbonate, calculated anion gap, and HbA1c levels were extracted from the electronic medical record (EMR). Estimated glomerular filtration rate (eGFR) was calculated from serum creatinine using the CKD-EPI creatinine-based 2021 equation [17].

Start and stop dates and indications for alpelisib, all antidiabetic therapies (metformin, SGLT2 inhibitors, dipeptidyl-peptidase 4 [DPP4] inhibitors, glucagon-like peptide receptor agonists [GLP1-RA], sulfonylurea, thiazolidinedione, meglitinide, and insulin), and corticosteroids (prednisone, hydrocortisone, methylprednisolone, and dexamethasone) were captured by manual chart review. Alpelisib interruptions, dose reductions, and discontinuations, along with the reason for these events, were also captured by manual chart review, as were dates and reasons for hospitalizations. All chart reviews were conducted by two reviewers and discrepancies in medication exposure dates were resolved by consensus among three members of the study team. Days spent inpatient (excluding the day on which hospitalization occurred) were excluded from the analysis because outpatient antidiabetic drugs are typically held and replaced with insulin during hospital admission.

Follow-up ended with permanent alpelisib discontinuation, death, loss to follow-up (defined as three or more months with no encounters in the MSK system), or on 6/30/2022.

The co-primary outcomes were change in random glucose levels (mg/dL) as measured from serum or point-of-care testing at MSK facilities, and incidence of DKA. Home glucose monitoring, including continuous glucose monitoring, was not captured. Rates of hospitalization due to hyperglycemia as well as alpelisib interruptions, dose reductions, and discontinuation due at least in part to hyperglycemia were reported along with a composite of all these events as ‘hyperglycemia-related treatment disruption.’ Rates of death and progression of disease were also reported. DKA was defined as events satisfying all of the following diagnostic criteria upon presentation: (1) serum bicarbonate ≤ 18 mmol/L and/or blood pH ≤ 7.3, (2) anion gap > 10 mEq/L, and (3) presence of serum and/or urine ketones.[18] These criteria were applied by two chart reviewers.

Rates of DKA were calculated, both overall and stratified into three exposure categories for patients on alpelisib: (1) no antidiabetic drugs, (2) on antidiabetic drugs excluding SGLT2 inhibitors, and (3) on SGLT2 inhibitors (with or without other antidiabetic drugs). Each case was also described with respect to initial symptoms, vital signs, laboratory data (eg, serum and point-of-care glucose levels), medications, clinical management, and outcome.

Change in blood glucose levels associated with each time varying exposure were described and analyzed using a mixed linear model, hierarchical at the patient level. The analysis of change in blood glucose level was restricted to time periods when patients were taking alpelisib. Patients on antidiabetic drugs at baseline were excluded from this specific analysis, as were patients who did not have random glucoses measured both at baseline and during follow-up. Antidiabetic drugs and corticosteroid exposure were included as time-varying variables (e.g., a patient who took metformin for only a portion of their follow-up time would be classified as metformin-exposed during that period, but not before or afterwards). Unadjusted changes in glucose level from baseline associated with exposure to each antidiabetic drug class and to corticosteroids are reported. In the primary adjusted analysis, antidiabetic drugs and steroid exposure were all included together in the mixed linear model as time-varying covariates. Results were further adjusted for baseline age, sex, date of alpelisib initiation, eGFR, glucose levels, and BMI.

In sensitivity analysis, to address concerns about adjustment for mediators, the mixed linear model was repeated as a marginal structural model (MSM), with stabilized inverse probability weights (IPW) applied to each period of follow-up. For example, if patients with no reduction of serum or point-of-care glucose levels after starting an SGLT2 inhibitor were likely to then stop SGLT2 inhibitor, this could create a bias in favor of SGLT2 inhibitor because non-responders to SGLT2 inhibitors would contribute less follow-up time. Including the initial change in glucose levels as a covariate would potentially adjust for this bias, but such adjustment could itself produce bias by ‘adjusting away’ the initial effects of SGLT2 inhibitor use on glucose levels, even though such an effect would be a mediator of SGLT2 inhibitor benefit, not a confounder. By using weights instead of covariates, an MSM is able to adjust for such potential mediators without producing bias.[19] MSM was conducted separately for metformin, SGLT2 inhibitors, and for corticosteroids. For each MSM, the IPW was calculated using exposure history to metformin, SGLT2 inhibitor, and corticosteroids as well as prior random glucose readings. Other antidiabetic drug classes were not included in the MSM because there were insufficient observations for the models to calculate IPW to converge. Models were also adjusted for non-time varying covariates measured at baseline: age, sex, date of alpelisib initiation, eGFR, glucose levels, and BMI.

Work in preclinical models has indicated that choice of antidiabetic drug may have implications not only for glucose levels on alpelisib, but on levels of insulin (or C-peptide). Differences in insulin level could be clinically significant in this context because high insulin levels could in theory override PI3K inhibition and undermine the anti-cancer effect of alpelisib and similar drugs. In an exploratory analysis, random C-peptide levels were also extracted from the electronic medical record and levels of C-peptide summarized in four exposure categories for patients: (1) not on alpelisib (ie, before or after exposure, since all study patients were on alpelisib at some point), (2) on alpelisib but no antidiabetic drugs, (3) on alpelisib with antidiabetic drugs excluding SGLT2 inhibitors, and (4) on alpelisib and SGLT2 inhibitor (with or without other antidiabetic drugs).

## Results

We identified 103 eligible patients who initiated treatment with alpelisib between October 23, 2019 and May 26, 2022 (Table 1). The median age of the cohort was 61 years (interquartile range [IQR] 55, 68). Of the patients, 102 (99%) were female, and 101 (99%) had metastatic breast cancer; two patients with ovarian cancer received alpelisib on a clinical trial protocol. In terms of race, 83 patients (81%) self-identified as White, eight (8%) as Asian, eight (8%) as Black, and four (4%) as other or were unknown; in terms of ethnicity, seven (7%) identified as Hispanic. Median baseline HbA1c was 5.5% (IQR 5.2, 5.8 [37 mmol/mol; IQR 33, 40]), in the 47 patients (46%) with available data. Eight patients (8%) had type 2 diabetes diagnosed at baseline. The median BMI was 25.6 (IQR 22.5, 28.9) and eGFR was 85 (IQR 76, 98). At the time of alpelisib initiation, six patients (6%) were already taking antidiabetic drugs (Table 1). Of those taking antidiabetic drugs, one was taking a meglitinide, four were taking metformin alone, and one was taking metformin and a DPP4 inhibitor.

After starting alpelisib, patients had median follow-up of 85 days (IQR 40, 212) (Table S1). Of the 103 patients, 63 (61%) permanently discontinued alpelisib during the follow-up period. Hyperglycemia was the primary or contributing cause in 11 of the 63 cases (17%). The median random glucose level prior to alpelisib initiation was 100 mg/dL (IQR 92,114). The mean follow-up glucose level after alpelisib treatment was 146 mg/dL. Metformin was prescribed to 34 patients (33%), SGLT2 inhibitor to 11 (11%), insulin to seven (7%), DPP4 inhibitor to three (3%), sulfonylurea to two (2%), and thiazolidinedione to two (2%) during alpelisib treatment. In the cohort, 21 patients (20%) experienced hyperglycemia-related treatment disruption, five (5%) presented with DKA, 36 (35%) experienced progression of disease and discontinued alpelisib, and 21 (20%) died.

**Figure 1:**
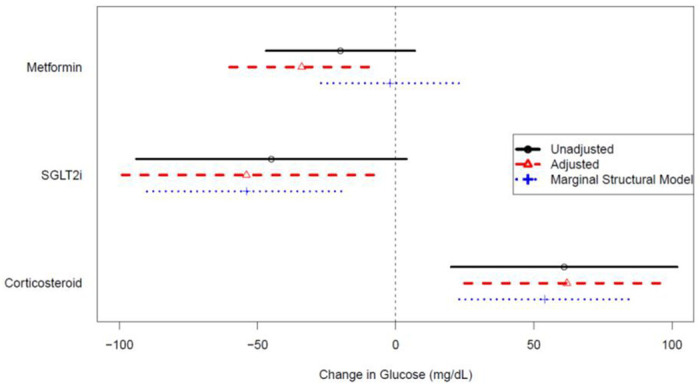
Association of time-varying drug exposures with change in mean random glucose with 95% confidence intervals; unadjusted (black solid line with circle), adjusted (red dashed line with triangle), marginal structural model (blue dotted line with cross).

In the analysis of the effect of antihyperglycemics on mean random glucose levels, 77 patients (75%) were included (after exclusion of six patients (6%) on antidiabetic drugs at baseline and 20 (20%) without glucose measurements during the follow-up period). An average of seven glucose readings over mean follow-up of 166 days were analyzed. In unadjusted mixed linear modeling, eight of 77 patients (10%) had time with SGLT2 inhibitor exposure, which was associated with lower mean glucose levels compared to SGLT2 inhibitor unexposed time in adjusted linear analysis (−54 mg/dL [95% CI −99 to −8]). Time with metformin exposure, as contributed by 31 of 77 patients (40%) who received metformin, was also associated with lower mean glucose levels in adjusted linear analysis (−34 mg/dL [95% CI −60 to −7]) (Fig. 1). Confidence intervals (CIs) for other antidiabetic drug classes were wide and crossed null in the setting of limited sample size: four patients (5%) received insulin, which had adjusted glucose reduction of −43 mg/dL (95% CI −111 to 28); two patients received a sulfonylurea (−4 mg/dL; 95% CI −94 to 92); two patients received a thiazolidinedione (−45 mg/dL; 95% CI −144 to 55), and two patients received a DPP4 inhibitor (−73 mg/dL; 95% CI −157 to 10). Corticosteroids were used in the treatment of 11 of 77 patients (14%) and were associated with significantly increased mean glucose values (+71 mg/dL; 95% CI 42 to 98). MSM showed an association between SGLT2 inhibitor exposure and glucose reduction but not for metformin (Fig. 1). MSM could not be carried out on other antidiabetic drugs because insufficient data were available to run the statistical models needed.

We identified five cases of DKA (Table 2). Of these, two occurred in patients on concomitant SGLT2 inhibitor and alpelisib (with one patient also on metformin and one patient also on a sulfonylurea), two occurred in patients receiving concomitant metformin and alpelisib, and one occurred in a patient on no antidiabetic drugs.

Patients receiving concomitant alpelisib and SGLT2 inhibitor had an incidence of 24 DKA cases per 100 patient-years (95% CI 6–80). In comparison, patients receiving alpelisib concomitant with non-SGLT2 inhibitor antidiabetic drugs (e.g., metformin) experienced seven DKA cases per 100 patient-years (95% CI 0.1, 34), and patients on alpelisib only experienced four DKA cases per 100 patient-years (95% CI 0.1, 21) (Table 3).

In an exploratory analysis, a total of 49 random (i.e., non-fasting) C-peptide levels were measured in 23 patients (22%). Serum glucose levels were measured simultaneously for 48 of the 49 C-peptides (Supplementary Fig. 1A). Twelve samples were collected when patients were not receiving alpelisib (median serum C-peptide level = 4.0 ng/mL; IQR 2.3,.1), 13 were measured during periods when patients received alpelisib without any antidiabetic drugs (median 9.9 ng/mL; IQR 4.1, 13.8), 14 were measured during periods when patients received concomitant alpelisib and antidiabetic drugs other than an SGLT2 inhibitor (median 10.6 ng/mL; IQR 7.7, 14.5), and 10 were measured during periods when patients received concomitant alpelisib and an SGLT2 inhibitor (4.6 ng/mL; IQR 4.0, 8.1). Unadjusted mixed linear modeling showed increased C-peptide levels in patients on alpelisib (*P* < 0.01) and alpelisib plus other antidiabetic drugs (*P* < 0.01), but not on alpelisib plus SGLT2 inhibitor (Supplementary Fig. 1B).

## Conclusions

Findings from this retrospective review show that SGLT2 inhibitor use is consistently associated with substantial reductions in glucose levels in patients on alpelisib. However, the occurrence of two cases of DKA in 11 patients (18%) on an SGLT2 inhibitor is concerning. SGLT2 inhibitors may be a uniquely effective antidiabetic drug class in patients on alpelisib, but the risk of DKA must be considered and mitigated. These findings may also be relevant to managing hyperglycemia in patients taking other drugs that inhibit the PI3K/AKT pathway.

The glucose-lowering effectiveness for SGLT2 inhibition in this study is consistent with previous animal data, clinical case reports, and exploratory observational analyses [9, 12, 14, 15]. This study’s strengths relative to previous work are that it is hypothesis testing rather than exploratory, controlled for important confounders (particularly steroid use), and includes sensitivity analysis using an MSM. This study was too small to establish whether SGLT2 inhibitors are more effective than other agents, such as metformin, in the setting of alpelisib use. However, SGLT2 inhibitors are now the only drug class with consistent evidence of glucose-lowering effectiveness in the context of patients receiving alpelisib.

Alpelisib increases C-peptide levels in patients, a marker of insulin production [20, 21]. Our data suggest that SGLT2 inhibitors may abrogate this effect. This finding is consistent with preclinical data showing that SGLT2 inhibitors, but not metformin, could prevent high endogenous insulin levels in animal models on PI3K inhibitors [3]. Researchers have speculated that SGLT2 inhibitors might improve clinical response to PI3K inhibition through this mechanism, since high insulin levels might be reactive to PI3K inhibition and allow tumors to escape the anticancer effect. The exploratory findings here support further investigation of that hypothesis.

These potential benefits from SGLT2 inhibitor use with alpelisib need to be weighed against the potential for harm. The two cases of DKA observed in patients treated with a combination of alpelisib and an SGLT2 inhibitor imply a rate of approximately one case per four person-years of exposure, far higher than the DKA rates of approximately one case per 1,000 patient-years seen in patients with type 2 diabetes who take an SGLT2 inhibitor [22]. While two cases comprise a small sample, it is supported further by our previous study, which identified one unambiguous case of DKA in 15 patients treated with an SGLT2 inhibitor while using PI3K or AKT inhibitors [9]. Prescribers who choose to use an SGLT2 inhibitor with alpelisib should be aware of this potential risk.

Limitations of this analysis include that the results regarding effectiveness are limited by small sample size and potential for time varying confounding. For example, the decision to start any antidiabetic drug might be accompanied by lifestyle modifications that are not captured in EMR data. Given uncertainty about the relative effectiveness of different antidiabetic drug classes in the unusual clinical setting of PI3K inhibition, continuous glucose monitoring may help optimize care and collect more robust data on the effectiveness of different drug classes. Finally, the hypothesis that SGLT2 inhibitors might have anticancer effects requires prospective study. Several relevant clinical trials are currently recruiting, including in “Alpelisib, Fulvestrant and Dapagliflozin for the Treatment of HR+, HER2−, PIK3CA Mutant Metastatic Breast Cancer” (NCT05025735) and TIFA (Targeting Insulin Feedback to Enhance Alpelisib: A Phase 2 Randomized Control Trial in Metastatic PIK3CA-mutant Hormone-Receptor Positive Breast Cancer [NCT05090358]).

Balancing the risks and benefits of SGLT2 inhibitors in patients on alpelisib is challenging. Due to the risk of DKA, clinical practice at our institution is not to use SGLT2 inhibitors as first-line agents, as many patients can achieve adequate glycemic control with metformin and lifestyle modification. However, SGLT2 inhibitors are our preferred second-line choice either in addition to or in place of metformin. We advise patients of the risk of DKA and that poor dietary intake is likely to increase the risk, so that they should hold the SGLT2 inhibitor on any day they are fasting or consuming less than 50% of their usual caloric intake. The value of home urine ketone monitoring is unclear because asymptomatic ketosis may be common on SGLT2 inhibitors and may not warrant intervention [23].

In summary, these study findings can aid in treatment decisions when managing PI3K inhibitor-induced hyperglycemia. While these findings apply to alpelisib specifically, they likely generalize to other PI3Kα inhibitors, pan-PI3K inhibitors, and potentially to other inhibitors on the PI3K/AKT pathway. We recommend that metformin and lifestyle modification should be the first-line treatment for alpelisib-induced hyperglycemia, given possible effectiveness for glucose lowering and a lack of serious adverse events. SGLT2 inhibitors are a reasonable second-line strategy with the caveat that providers should monitor for the development of DKA. Further research on the comparative effectiveness of other antidiabetic drug classes is warranted.

## Figures and Tables

**Table 1:** Baseline characteristics

Characteristics at baseline	*N* = 103*
Age, years	61 (55, 68)
Female sex	102 (99)
Race	
Asian	8 (8)
Black	8 (8)
Other or unknown	4 (4)
White	83 (81)
Hispanic ethnicity	7 (7)
Diagnosis	
Metastatic breast cancer	101 (99)
Ovarian cancer	2 (2)
Baseline antidiabetic drug use	6 (6)
Type 2 diabetes	8 (8)
Cardiovascular disease	31 (30)
Hypertension	42 (41)
Renal disease	2 (2)
Liver disease	12 (12)
Anemia	20 (19)
Random glucose, mg/dL	100 (92, 114)
Estimated glomerular filtration rate	85 (76, 98)
HbA1c, %, mmol/mol	5.5 (5.2, 5.8), 37 (33, 40)
Missing	56 (54)
Albumin, g/dL	4.03 (3.80, 4.30)
Serum bicarbonate, mmol/L	25.00 (23.00, 27.00)
Anion gap, mEq/L	10.00 (9.00, 11.00)
Body mass index, kg^2^/m	25.6 (22.5, 28.9)

* Except where noted, data are presented as median (IQR); n (%)

**Table 2:** Cases of diabetic ketoacidosis (DKA)

	Case 1	Case 2	Case 3	Case 4	Case 5
**Antidiabetic regimen**	Metformin + SGLT2	Metformin	Sulfonylurea + SGLT2	None	Metformin
**Days on alpelisib**	123	20	43	8	14
**Days on SGLT2 inhibitor**	10	-	31	-	-
**Blood glucose**	147	581	357	361	350
**Serum bicarbonate, mmol/L**	16	14	16	17	18
**Anion gap, mEq/L**	23	21	16	19	15
**Venous blood gas**	7.36	7.31	7.35	7.28	7.33
**Urine/serum ketones**	+	+	+	+	+
**DKA presentation**	Called endocrine service with asymptomatic elevation in urine ketones at home	Presented to clinic with polyuria, weight loss, decreased appetite, and altered sense of taste	Presented to ED with poor oral dietary intake, emesis	Presented to ED with diarrhea, fall, rash	Presented to ED with hyperglycemia, polyuria, polydipsia
**Treatment**	Insulin drip	Insulin drip	Insulin drip	Basal-bolus insulin	Basal-bolus insulin
**Outcome**	Stopped SGLT2 inhibitor; continued alpelisib; managed on DPP4 inhibitor and sulfonylurea until progression of disease, when alpelisib was stopped	Stopped alpelisib; discharged on insulin, metformin, and DPP4 inhibitor	Stopped SGLT2 inhibitor; continued alpelisib; discharged on sulfonylurea and DPP4 inhibitor; readmitted with recurrent DKA 5 days later; rapid progression of disease, deceased	Stopped alpelisib; rapid progression of disease, deceased	Continued alpelisib; discharged on insulin, metformin

ED, emergency department

**Table 3:** Incidence of diabetic ketoacidosis.

Medication	N*	Person-Days	DKA cases	DKA / 100 patient-years (95% confidence interval)
**Alpelisib only**	91	8964	1^†^	4 (0.1, 21)
Alpelisib + non-SGLT2 inhibitor Antihyperglycemic drugs	44	5025	2^†^	7 (0.1, 34)
Alpelisib + SGLT2 inhibitor	12	1548	2	24 (6, 80)

* Total exceeds 103 because many patients contributed time to multiple exposure categories

† Includes one case with plausible alternative explanations for laboratory values (including starvation, renal failure, and diarrhea)

## Data Availability

The datasets generated during and/or analyzed during the current study are available from the corresponding author on reasonable request.
